# Mexican Oregano (*Lippia berlandieri* Schauer and *Poliomintha longiflora* Gray) Essential Oils Induce Cell Death by Apoptosis in *Leishmania (Leishmania) mexicana* Promastigotes

**DOI:** 10.3390/molecules27165183

**Published:** 2022-08-15

**Authors:** Karla Fabiola Chacón-Vargas, Luvia Enid Sánchez-Torres, Mónica L. Chávez-González, Jaime R. Adame-Gallegos, Guadalupe Virginia Nevárez-Moorillón

**Affiliations:** 1 Facultad de Ciencias Químicas, Universidad Autónoma de Chihuahua, Circuito Universitario S/N, Chihuahua 31125, Mexico; 2 Escuela Nacional de Ciencias Biológicas, Instituto Politécnico Nacional, Prolongación de Carpio y Plan de Ayala S/N, Mexico City 11340, Mexico; 3 Bioprocesses and Bioproducts Research Group, Food Research Department, School of Chemistry, Universidad Autónoma de Coahuila, Saltillo 25280, Mexico

**Keywords:** leishmanicidal, *Lippia berlandieri*, *Poliomintha longiflora*, essential oils

## Abstract

Leishmaniasis is a neglected vector-borne disease; there are different manifestations of the diseases and species involved, and cutaneous leishmaniasis caused by *Leishmania (L.) mexicana* is the most prevalent in Mexico. Currently, the drugs available for the treatment of leishmaniasis are toxic, expensive, and often ineffective; therefore, it is imperative to carry out research and development of new therapeutic alternatives, with natural products being an attractive option. In particular, oregano is a plant with worldwide distribution; in Mexico, two species: *Lippia berlandieri* Schauer and *Poliomintha longiflora* Gray are endemic. Both essential oils (EO’s) have been reported to have antimicrobial activity attributed to their main components, thymol and carvacrol. In this research, the leishmanicidal effect and mechanism of cell death induced by *L. berlandieri* EO, *P. longiflora* EO, thymol, and carvacrol in *L. mexicana* promastigotes were determined in vitro. Additionally, the cytotoxic activity in mammalian cells was evaluated. *L. berlandieri* EO presented higher leishmanicidal activity (IC_50_ = 41.78 µg/mL) than *P. longiflora* EO (IC_50_ = 77.90 µg/mL). Thymol and carvacrol were the major components of both Mexican oregano EO’s. Thymol presented higher leishmanial inhibitory activity (IC_50_ = 22.39 µg/mL), above that of carvacrol (IC_50_ = 61.52 µg/mL). All the EO’s and compounds evaluated presented lower cytotoxic activity than the reference drug; thymol was the compound with the best selectivity index (SI). In all cases, apoptosis was identified as the main mechanism of death induced in the parasites. The leishmanicidal capacity of the Mexican oregano EO is an accessible and affordable alternative that can be further explored.

## 1. Introduction

Leishmaniasis is a vector-borne disease caused by different species of protozoa of the genus *Leishmania*, and they are transmitted to humans and animals through the bite of infected female sandflies of the *Lutzomyia* genus in the Americas [1]. The disease is directly linked to poverty, host immune status, and environmental and climatic factors influencing its epidemiology; the WHO considers leishmaniasis a neglected tropical disease (NTD) [2,3]. It is estimated that more than 12 million people are infected worldwide, with an average of 1.3 million new cases, 20 to 30 thousand deaths, and 350 million people at risk of infection per year [1,4].

The clinical manifestations of leishmaniasis are classified as cutaneous (CL), mucocutaneous (ML), or visceral (VL). CL is the most common presentation and causes lifelong scars, ML causes partial or complete nose and mouth mucous membrane destruction and can lead to disability, and VL presents irregular episodes of fever, weight loss, hepatosplenomegaly, and anemia and can cause death in more than 90% of untreated cases [4]. In Mexico, the protozoan *Leishmania (Leishmania) mexicana* is endemic in the central–southwestern region of the country; it mainly causes CL, affecting the ear and face of workers in the forest in most cases (popularly known as Chiclero’s ulcer) [5,6].

The treatment for leishmaniasis is chemotherapy; pentavalent antimonials are the first-line drugs, in addition to amphotericin B, miltefosine, pentamidine, and paromomycin as second-line drugs. Unfortunately, the available treatments are strongly related to high toxicity, multiple side effects, and long administration periods that limit their clinical use and favor non-adherence to treatment [7,8]. Nowadays, it is necessary to investigate new therapeutic alternatives; some research groups have directed the search for new compounds with biological activity toward natural sources such as the extracts and essential oils (EOs) of medicinal and aromatic plants with no or minimal adverse effects. There are many endemic species in Mexico, and an ancient tradition is deeply rooted in their medicinal use [9].

Oregano is a plant of worldwide distribution; in Mexico, at least 40 endemic species are known. *Lippia berlandieri* Schauer or *Lippia graveolens* Kunth (*Verbenacea* family) are important culinary ingredients and the most important economically, having a wide distribution in the arid and semi-arid areas of the country [10]. *Poliomintha longiflora* Gray (*Laminacea* family) is another species traded as “Mexican oregano” and grows wild in the northeast of the country [11]. Oregano chemotypes have been identified based on the concentration of their main components: thymol and carvacrol [12]. The EOs of *L. berlandieri* and *P. longiflora* have been chemically characterized, and their biological activity has been widely studied and reported by our research group [11,12,13,14,15,16,17]. The antimicrobial properties are mainly attributed to their major components, thymol and carvacrol [18]; their effectiveness may vary by the EO composition and the microorganisms cellular structure [17]. 

There are some reports of the parasiticidal effect of the EOs of oregano on different species of *Leishmania*. [19,20,21]. Recently, we reported the biological evaluation of several EOs in different microorganisms, including the effect of Mexican oregano *Lippia berlandieri* EO on *L (L.) mexicana*; our results showed its leishmanicidal activity by inhibiting cell metabolism [17]. However, it is necessary to perform a complementary analysis to characterize the induced death mechanism. The purpose of this research was to characterize the effect induced by two Mexican oregano EOs, *Lippia berlandieri* Schauer and *Poliomintha longiflora* Gray, as well as their major components, thymol and carvacrol, on the protozoan *L. mexicana* to identify the specific cellular damage caused and the type of cell death induced, as a first step to elucidate the mechanism of action and to determine their therapeutic targets.

## 2. Results 

### 2.1. Chemical Composition of Essential Oils of Mexican Oregano

The two essential oils (EOs) of Mexican oregano were analyzed to determine the relative concentration of the carvacrol and thymol. The chemical characterization of the EOs was performed by gas chromatography/mass spectrometry [16]. Thymol and carvacrol were the most abundant components in both EOs; however, *L. berlandieri* had a higher percentage of relative concentration of carvacrol (33.78%) than thymol (7.86%). On the other hand, *P. lonfliglora* presented a higher relative concentration of thymol (23.46%) than carvacrol (18.35%) (Table S1).

### 2.2. Leishmanicidal and Cytotoxic Activity of Essential Oils of Mexican Oregano and Their Two Major Components

The evaluation of the biological activity of the EOs of Mexican oregano *Lippia berlandieri* and *Poliomintha longiflora* as well as their pure major components, thymol and carvacrol, on the metabolism of promastigotes of *L. mexicana* was carried out by the fluorometric method with Alamar Blue. Different concentrations were evaluated to determine the 50% inhibitory concentration (IC_50_) of each. Both EOs and the pure compounds showed a concentration-dependent inhibitory effect; *L. berlandieri* presented the best leishmanicidal activity of both essential oils (IC_50_ = 41.78 µg/mL) (Table 1). Regarding the evaluation of the pure major components, thymol presented a higher inhibitory activity, with an IC_50_ = 22.39 µg/mL, than carvacrol (IC_50_ = 61.52 µg/mL) and both essential oils. In contrast, the EOs of *P. longiflora* presented the lowest effect (IC_50_ = 77.90 µg/mL). Amphotericin B (IC_50_ = 0.41 µg/mL) was used as a reference drug, and none of the compounds exceeded its in vitro leishmanicidal activity.

The cytotoxic effect in murine macrophage cell line was evaluated, and 50% cytotoxic concentration (CC_50_) was determined. The *P. longiflora* EO presented the lowest cytotoxic effect (CC_50_ = 166.66 µg/mL), followed by *L. berlandieri* (CC_50_ = 150.41 µg/mL) and carvacrol (CC_50_ = 147.37 µg/mL), with similar results among them. On the other hand, thymol exhibited the highest cytotoxic effect (CC_50_ = 114.13 µg/mL). Interestingly, the four substances had less cytotoxic effect than Amphotericin B (CC_50_ = 112.45 µg/mL).

Finally, the selectivity indexes (SI = CC_50_/IC_50_) for both EOs of Mexican oregano, thymol and carvacrol were determined. Thymol was the most selective compound with an SI = 5.10. The selectivity index reflects the impact of the compound on the microorganism. For oral administration, an SI ≥ 10 is considered adequate. In this case, both thymol and carvacrol showed SI < 10, suggesting that topical administrations are a better option for further in vivo evaluations [22].

### 2.3. Pharmacodynamic Interaction of Thymol and Carvacrol

The possible interaction of the leishmanicidal effect between thymol and carvacrol was determined in vitro by combining different concentrations. Thirty-six different combinations of thymol and carvacrol were evaluated from six concentrations of each (200, 100, 50, 25, 12.5, 6.25 µg/mL). The fractional inhibitory concentration (FIC) values obtained with the different combinations of monoterpenes concentrations ranged from FIC = 0.75 to FIC = 1.13, where the average was FIC = 0.92. The latter value indicates an additive leishmanicidal effect between thymol and carvacrol at different concentrations [23]. Some other authors omit the term “additive interaction”, and they classify the result obtained in the range of “indifference effect (FIC = 0.5–4)” [24,25]. However, in both cases, the interpretation is the same, and the combination of thymol and carvacrol lacks antagonistic or synergistic effect; they can be used alone or in combination and their leishmanicidal effect does not change.

### 2.4. Metabolic Inhibition and Loss of Cell Membrane Integrity

After confirming the metabolic inhibitory capacity of EOs and thymol and carvacrol in promastigotes of *L. mexicana,* we were interested in knowing their effect on the integrity of the parasite plasma membrane. Propidium iodide (PI) is an impermeable compound that binds to DNA. In non-viable cells with membrane rupture, the fluorochrome diffuses into the cell and intercalates with DNA, showing positive fluorescence in a flow cytometer; in cells with an intact plasma membrane, the fluorescence is negative [26].

**Figure 1:**
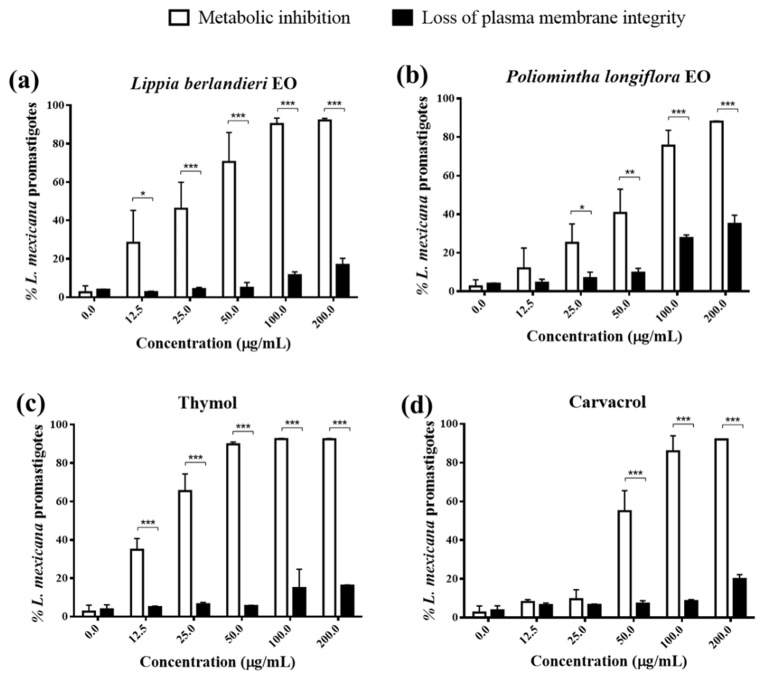
(**a**) Effect of *Lippia berlandieri* EO, (**b**) *Poliomintha longiflora* EO, (**c**) thymol, and (**d**) carvacrol on metabolic inhibition and loss of plasma cell membrane integrity of *Leishmania mexicana* promastigotes. Parasites were treated with 0, 12.5, 25, 50, 100, and 200 μg/mL of EOs or pure compounds and incubated for 24h. Each bar represents the mean ± standard deviation of three experiments (*n* = 3). Statistical comparison was performed with two-way ANOVA (concentration x effect) with Sidak’s multiple comparison test, * *p* ≤ 0.05, ** *p* ≤ 0.01, and *** *p* ≤ 0.001.

Figure 1 compares the effect of *L. berlandieri* EO, *P. longiflora* EO, thymol, and carvacrol on the parasite’s metabolic inhibition and plasma membrane integrity at different concentrations. In most cases, the percentage of parasites with metabolic inhibition is higher than the percentage of parasites with loss of plasma membrane integrity; this difference is most marked at higher concentrations. To both EOs and pure compounds, the 50% lytic concentration (LC_50_), or concentration that causes plasma membrane rupture of 50% of the parasites, is greater than 200 µg/mL, highlighting that LC_50_>>IC_50._

Accordingly, the EOs of Mexican oregano and the pure compounds can promote the inhibition of the metabolism in the promastigotes of *L. mexicana* while maintaining the integrity of the plasma membrane even at high concentrations. This phenomenon suggests the ability to induce a cell death process by apoptosis. To evaluate this hypothesis, we proceeded to analyze the morphological changes and different markers of cell death induced by the EOs, thymol, and carvacrol.

### 2.5. Effects on Cellular Morphology

To evaluate the effect of the EOs of *L. berlandieri, P. longiflora*, thymol, and carvacrol on the cellular morphology of *L. mexicana* promastigotes, two strategies were used: structural analysis by light microscopy and changes in size by flow cytometry.

**Figure 2:**
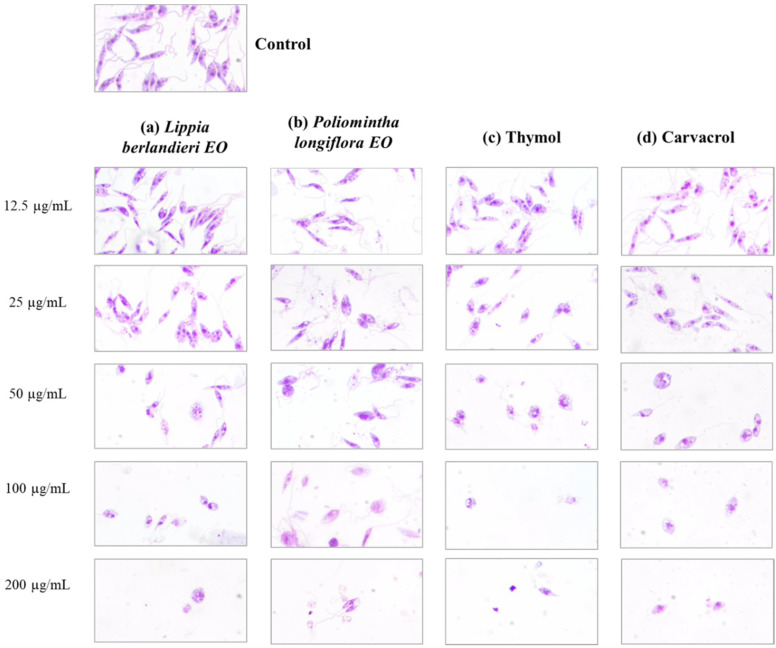
Effect of (**a**) *Lippia berlandieri* EO, (**b**) *Poliomintha longiflora* EO, (**c**) thymol, and (**d**) carvacrol on morphological changes in *Leishmania mexicana* promastigotes. Parasites were treated with 0, 12.5, 25, 50, 100, and 200 μg/mL of EOs or pure compounds, incubated for 24h, stained with Giemsa, and analyzed by light microscopy at 100X.

The analysis by light microscopy revealed that the compounds induced morphological changes compared with the morphology of the untreated promastigotes. In control promastigotes (0 µg/mL), the typical morphology of *L. mexicana* promastigotes showed elongated cells with well-defined flagellum, nucleus, and kinetoplast (Figure 2).

Regarding the treated promastigotes, a concentration-dependent effect was observed for the EOs and pure compounds. No morphological changes were identified at the lowest concentration (12.5 µg/mL) compared to the untreated control. At 25 µg/mL, a slight rounding in the posterior portion of the parasites was observed; this effect was more evident as concentration increased. In the case of *L. berlandieri* EO and *P. longiflora* EO, a mixture of rounded and elongated cells was observed at 50 µg/mL. At the highest concentrations, cell density decreased drastically, and the cells were mainly rounded and shrunken, without definition of the organelles and with retraction of the flagellum. 

**Figure 3:**
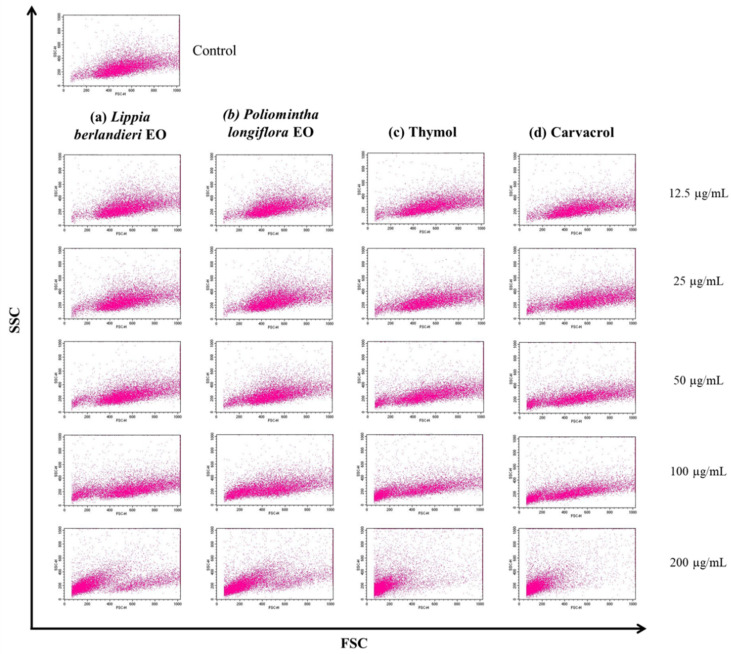
Effect of (**a**) *Lippia berlandieri* EO, (**b**) *Poliomintha longiflora* EO, (**c**) thymol, and (**d**) carvacrol on size (FSC) and granularity (SSC) of *Leishmania (L.) mexicana* promastigotes. Parasites were treated with 0, 12.5, 25, 50, 100, and 200 μg/mL of EOs or pure compounds, incubated for 24 h, and analyzed by flow cytometry. Images are representative of three independent experiments.

The morphological alterations were consistent with the changes in size obtained by flow cytometry. Figure 3 shows the distribution of untreated parasites (0 µg/mL), with a central population in the FSC vs. SSC dot plot, a characteristic pattern of viable cells. In the promastigotes treated with the different concentrations of EOs and the pure compounds, a gradual decrease in size (FSC) and complexity (SSC) was observed, and cells with reduced size and complexity were grouped at the origin of the graph; a concentration-dependent effect was confirmed. At the highest concentration, 200 µg/mL, thymol and carvacrol showed a more significant effect by reducing the size of most of the cell population with respect to *L. berlandieri* EO and *P. longiflora* EO.

In Figure 3, a decrease in cell density is not appreciated because the same number of cells were acquired per sample. However, in the evaluation at the highest concentrations, the acquisition time rose considerably due to the decreased number of cells present in the sample.

### 2.6. Characterization of the Mechanism of Induced Cell Death

According to the previous results, it was demonstrated that *L. berlandieri* EO, *P. longiflora* EO, and their major components thymol and carvacrol induced inhibition of metabolism with preservation of the integrity of the plasma membrane in *L. mexicana* promastigotes (Figure 1) and size reduction (Figure 2 and Figure 3). Subsequently, we performed a complementary analysis to determine whether the mechanism of cell death induced in the parasite is apoptosis, as suggested above. The parasites were exposed to five concentrations for 24 h, and later, different cell death markers were evaluated by flow cytometry.

**Figure 4:**
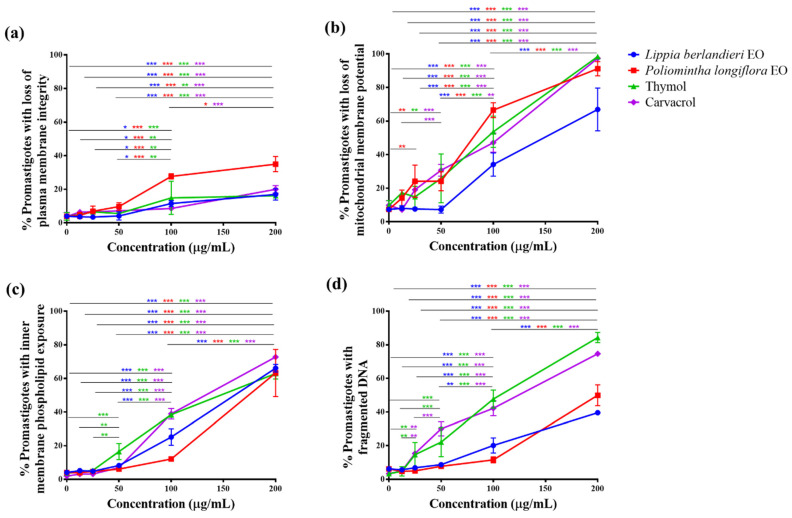
Characterization of the effect induced on *Leishmania mexicana* promastigotes by *Lippia berlandieri* EO, *Poliomintha longiflora* EO, thymol, and carvacrol. Parasites were treated with 0, 12.5, 25, 50, 100, and 200 μg/mL of EOs, thymol, or carvacrol; incubated for 24 h; and stained and analyzed by flow cytometry. (**a**) Loss of plasma membrane integrity, (**b**) loss of mitochondrial membrane potential, (**c**) inner membrane phospholipids (PL) exposition, and (**d**) DNA fragmentation. Data represent the mean and SD of three experiments. Statistical comparison was performed with two-way ANOVA (concentration x effect) with Tukey’s multiple comparison test, * *p* ≤ 0.05, ** *p* ≤ 0.01, and *** *p* ≤ 0.001.

As mentioned, the evaluation of the integrity of the plasmatic membrane was carried out using PI fluorochrome. Figure 4a reports the percentage of parasites with loss of plasma membrane integrity at different concentrations. A concentration-dependent effect was observed; *L. berlandieri* EO, thymol, and carvacrol reached around 20% of parasites with loss of plasma membrane integrity at the highest concentration (200 µg/mL). Regarding the effect of *P. longiflora* EO, the number of cells with membrane damage in the plasma membrane at both highest concentrations (100 and 200 µg/mL) is almost twice the others, which suggests that it is due to the combined effect of all the essential oil components.

The mitochondrial membrane potential (ψ_m_) was evaluated with the cationic fluorochrome Rhodamine 123 (Rho 123), which accumulates in the mitochondria of viable cells due to the negative mitochondrial membrane potential. EOs, thymol, and carvacrol showed a gradual loss of mitochondrial membrane potential in a concentration-dependent way. The results obtained from *P. longiflora* EO, thymol, and carvacrol presented a similar tendency (Figure 4b); at the lowest concentrations (12.5 and 25 µg/mL), most parasites (>80%) showed active mitochondrial membrane potentials. However, at 100 µg/mL (>IC_50_), about 50% of cells lost mitochondrial membrane potential, and finally, at the highest concentration, 200 µg/mL, most cells (>90%) showed no mitochondrial activity. Mitochondria were less affected by *L. berlandieri* EO as 200 µg/mL caused alteration in the mitochondrial membrane potential in 67% of the parasites. The results suggest that the mitochondrion is a vulnerable organelle for the compounds and EOs of interest.

Phosphatidylserine (PS) is a phospholipid found on the inner side of the plasma membrane of eukaryotic cells. When there is a membrane rupture caused by necrotic death or translocation of PS by apoptotic death, PS can be identified by the binding of the annexin V molecule (AnnV) coupled to a fluorochrome. In protozoa such as *Leishmania* spp., Weingärtner et al. described that PS could be absent, but the presence of other anionics phospholipids (FL) responsible for the Ann V binding effect observed in cell death processes was observed [27]. It has been reported that phosphatidylcholine (FC) is the most abundant phospholipid in *Leishmania* spp [27,28]. Figure 4c shows that EOs and both pure compounds induced the exposure of internal anionic phospholipids as the concentration increased; at 100 µg/mL, thymol and carvacrol presented greater effects than the EO’s.

DNA fragmentation is considered a hallmark of cell death. The parasites treated were permeabilized and stained with a PI/RNAse solution for flow cytometric analysis. The fluorescence intensity is proportional to the stoichiometric binding of fluorochrome to nucleic acids; the different phases of the cell cycle and cells with DNA fragmentation (Sub-G0 peak) due to cell death were identified. With a concentration-dependent effect, *L. berlandieri* EO, *P. longiflora* EO, thymol, and carvacrol induced DNA fragmentation (Figure 4d). The pure compounds thymol and carvacrol induced a higher percentage of cells with fragmented DNA from 50 µg/mL.

The integration of the evaluation results of the different parameters confirmed that the leishmanicidal effect of the *L. berlandieri* EO and *P. longiflora* EO is due to the biological activity of its major components (Figures S1 and S2, respectively). Thymol and carvacrol induced cell death by the apoptosis mechanism, with a reduction in cell volume, loss of mitochondrial membrane potential, exposure of internal membrane phospholipids, and DNA fragmentation but with a maintaining of the integrity of the plasma membrane (Figures S3 and S4, respectively). The induced cell death by the pure compounds is maintained in both EOs despite the presence of other components.

## 3. Discussion

The results obtained in this study show that two EOs of Mexican oregano have potential leishmanicidal activity. *L. berlandieri* EO and *P. longiflora* EO inhibited the growth of *L. mexicana* promastigotes. The terpenes thymol and carvacrol are the main components in both EOs; however, their proportions and effect were different. The chemical characterization showed that 41.64% of *L. berlandieri* EO is constituted by thymol and carvacrol, where carvacrol is the most abundant (33.78%). In addition to the leishmanicidal activity, the antimicrobial activity of the carvacrol-rich chemotype of *L. berlandieri* EO has previously been reported [12,16]. The EO of *L. berlandieri* is characterized by its flavor and pungent smell due to its higher content of carvacrol; this characteristic is quality-related and is the most commercialized and exported species in Mexico. 

In the EO of *P. longiflora*, thymol and carvacrol accounted for 41.81% of its composition (similar to *L. berlandieri* EO); however, the most abundant compound was thymol (23.46%). *P. longiflora* EO has a milder flavor due to its low carvacrol content; for this reason, it is commonly used to substitute European oregano (*Origanum vulgare*) in the culinary field [29]. 

For *Leishmania (L.) mexicana* promastigotes, the *L. berlandieri* EO was more effective (IC_50_ = 41.78 µg/mL) that *P. longiflora* EO (IC_50_ = 79.70 µg/mL). The leishmanicidal activity of different EOs of *Lippia spp.* has been documented. The inhibitory effect of the different chemotypes of *Lippia gracilis* EO and its effect on promastigotes of *Leishmania chagasi* has been reported; the carvacrol-rich chemotype (IC_50_ = 77.26 µg/mL) was more active than the thymol-rich chemotype (IC_50_ = 86.32 µg/mL) [30]. Similarly, carvacrol-rich *Lippia sidoides* EO (IC_50_ = 54.8 µg/mL) was more effective on *Leishmania chagasi* promastigotes than the thymol-rich chemotype (IC_50_ = 74.1 µg/mL) [31]. In other reports, the thymol-rich chemotype also presented leishmanicidal activity. Thymol-rich *Lippia origanoides* EO showed inhibitory effect in *L. chagasi* promastigotes (IC_50_ = 4.4µg/mL) [21], and thymol-rich *L. sidoides* EO presented significant activity against promastigotes of *L. chagasi* (IC_50_ = 89 µg/mL) [19] and *L. amazonensis* (IC_50_ = 44.38 µg/mL) [32]. 

Interestingly, ethanolic extracts of *L. sidoides* showed no leishmanicidal activity in *L. amazonensis* promastigotes [20], suggesting that the EO is more effective than the extracts, since there are more components and the concentrations are different.

In the evaluation of the pure compounds, *L. mexicana* promastigotes were more susceptible to thymol (IC_50_ = 22.39 µg/mL) than carvacrol (IC_50_ = 61.52 µg/mL); the same occurred in previous reports for *Leishmania infantum* [33]. In contrast, *Leishmania chagasi* was more sensitive to carvacrol than thymol [30,31], suggesting that the selectivity of terpenes depends the *Leishmania* species involved. 

The cytotoxicity evaluation was carried out in mammalian macrophages because they are the target cells for *Leishmania* spp. infection in vertebrates as intracellular amastigotes [34]. Both EOs and the pure compounds presented less cytotoxic effect than the reference drug, but thymol exhibited the highest cytotoxic effect. Other investigations have also reported the cytotoxic effect of thymol, which was evaluated in peritoneal macrophages and *L. amazonensis* promastigotes and presented a low SI. By contrast, when *L. sidoides* EO was evaluated, no cytotoxic effect was observed, and there was a notable decrease in intracellular amastigotes in infected macrophages [32]. This suggests that the interactions between the rest of the components present in *L. berlandieri* EO, *P. longiflora* EO, or *L. sidoides* EO reduced the cytotoxic effect of thymol.

Thymol and carvacrol are isomeric terpenes, and they differ in the position of the hydroxyl group in the aromatic ring concerning the isopropyl group [35]. In this report, thymol has better biological activity than carvacrol. The presence of the hydroxyl group in the ortho position reduces almost three times the leishmanicidal activity with respect to carvacrol with the hydroxyl group in the meta position; the cytotoxic effect is less affected, favoring an acceptable SI. 

The EOs of Mexican oregano and the pure compounds thymol and carvacrol presented a low IS (<10), being discarded for in vivo evaluations by enteral or parenteral routes. However, due to the high lipophilicity of the terpenes, topical or transcutaneous administration can be considered [36]. The demonstration of a local effect favors the treatment of cutaneous leishmaniasis caused by *L. mexicana* and others.

Topical application of thymol can be considered a good alternative of natural origin and with local effect that is easy to apply, convenient, economical, and safe for the patient. In addition, antibacterial properties of terpenes have been described [35] that would favor the treatment of bacterial infections associated with leishmaniasis ulcers. According to the above, thymol has advantages over first-line drugs, such as pentavalent antimonials, or second-line drugs, such as Amphotericin B. Finally, terpene molecules, mainly thymol, serve as a structural basis for the rational design of more effective and safer molecules with lower toxicity.

Thymol was the molecule with the highest leishmanicidal activity; although the *P. longiflora* EO has a higher proportion of thymol, it showed less biological activity than the *L. berlandieri* EO (carvacrol rich). When thymol and carvacrol are in the EO mixture, their effect is modified by one or more of the remaining components. The leishmanicidal effect of the Mexican oregano EOs varies according to the number and proportion of their main compounds and the rest of their components. They were indifferent when evaluating the pharmacological interaction between thymol and carvacrol. Therefore, the EOs of *L. berlandieri* and *P. longiflora* presented a greater leishmanicidal effect than the mixture of thymol and carvacrol in different proportions. EOs tend to have higher activity than mixtures of their major components, suggesting that minor components are critical for synergistic activity, although antagonistic and additive effects have also been observed [37].

The mechanism of action of EOs on protozoa has not been fully elucidated. In addition to the evaluation of metabolic inhibition, some studies reported ultrastructural analysis. In the case of *L. sidoides* EO, it induced drastic morphological changes in promastigotes of *L. chagasi* and *L. amazonensis,* such as cellular swelling, lipid accumulation, wrinkled cells, and increased volume of the acidocalcisoma [19,32]. The evidence of morphological changes is important. However, completing the analysis with the identification of the mechanism of cell death induced in the parasite by leishmanicidal compounds provides valuable information on metabolism and targets in the parasite to elucidate the mechanism of action [38,39]. 

The ability of the essential oils of Mexican oregano, thymol and carvacrol, to trigger changes associated with apoptosis in promastigotes of *L. mexicana* was notable. Initially, regulated cell death (RCD) was thought to occur only in multicellular organisms; however, a RCD process has been shown to also occur in unicellular eukaryotic organisms such as trypanosomatids [40]. 

During apoptosis, similar morphological changes occur in unicellular and multicellular organisms in response to different stimuli. In 2019, Basmaciyan and Casasola described “apoptosis” as a cell death phenotype and suggested avoiding the terms “apoptosis-like” or “programmed cell death”; in addition, they proposed unified criteria for the decision of “apoptosis” in *Leishmania* protozoa. They proposed two stages; initially, they demonstrated parasite death by assessing the loss of plasma membrane integrity by PI staining. As the second stage, for the characterization of apoptosis, they suggest the detection of two or more of the following markers: DNA fragmentation, cell rounding, cell shrinkage, plasma membrane modifications, and mitochondrial depolarization [40].

Our results demonstrated that *L. berlandieri* EO, *P. longiflora* EO, thymol, and carvacrol induced apoptosis cell death in *L. mexicana* promastigotes according to the above criteria. In the first stage, we determined that EOs and the major compounds induced the inhibition of cellular metabolism with the preservation of the integrity of the plasmatic membrane. The metabolic inhibition was determined by the fluorometric method with Alamar Blue and the integrity membrane with fluorochrome PI by flow cytometry. Figure 1 shows the increasing metabolic inhibition concerning concentrations as well as the low percentage of cells with loss of plasma membrane integrity. It is important to note that when the effect is necrotic, a high percentage of PI-positive parasites are observed [41,42].

In the second stage, EOs, thymol, and carvacrol presented more than two of the criteria considered to confirm apoptosis. The treated parasites presented cell rounding and shrinkage, inner plasma phospholipids exposition, mitochondrial membrane depolarization, and DNA fragmentation; all effects were concentration-dependent (Figure 4, Figures S1–S4). Other reports have also documented the induction of apoptosis death in *Leishmania spp.* due to the effect of different EOs [43,44].

The mechanism of action of EOs is unknown. They have a large number of chemical compounds, and their pharmacological action is not likely attributable to a single specific mechanism, but the proportions of the components and their interactions are responsible for the final biological activity [45]. The biological activity of thymol and carvacrol followed the same pattern as the EOs, suggesting that the rest of the components do not modify the cell death mechanism induced by the major compounds but only modify the potency of the effect.

Regarding the mechanism of action of monoterpenes (including citral, geraniol, linalool, carvacrol, p-cymene, eucalyptol, and eugenol, among others), it has been reported that they can cause structural and functional alterations in eukaryotic cells. Monoterpenes have high lipophilicity and low density; they can diffuse rapidly across cell membranes and accumulate inside cells. Subsequently, they cause apoptosis with plasma membrane destabilization, mitochondrial membrane permeability damage, and DNA fragmentation [33,46].

Additionally, it has been reported that monoterpenes can induce apoptosis in many types of cancer cells. The proposed mechanism involves several processes such as interaction and inhibition of mitochondrial membrane potential, impaired ATP synthesis, and DNA fragmentation [47]. The suggested mechanism of action of thymol and carvacrol in protozoa and cancer cells is similar because they are eukaryotic cells with the purpose of multiplication. The results obtained coincide with those reported in the literature [37,48], where it is suggested that thymol and carvacrol are internalized and accumulate in *L. mexicana* promastigotes. The initial effects are related to alterations in the mitochondrial membrane potential, triggering changes in the redox ratio, effects on the cell membrane and cell morphology, and DNA fragmentation, leading to apoptosis death. The final event is a late apoptosis, in which the cellular architecture is destabilized by rupturing the cytoplasmic membrane, increased permeability, and the release of metabolites leading to the destruction of the parasite [37,48].

## 4. Materials and Methods

### 4.1. Essential Oils and Pure Compounds

Mexican oregano, *Lippia berlandieri* Schauer, and *Poliomintha longiflora* Gray, were provided by the Center of Research for Natural Resources (CIReNa) Municipality of López, Chihuahua, Mexico. EOs of *L. berlandieri* and *P. longiflora* were obtained by steam distillation and characterized as described by Zapien-Chavarria et al. [16]; a Varian (CP3800) gas chromatography coupled to a mass spectrometer (Saturn 2000, USA), using an Agilent J&W 5MS column (30 m × 0.25 mm I.D. × 0.25 µm), which was used for the analysis. Helium was used as the carrier gas, at 1 mL/min flux, and the following conditions were used for the analysis: a 1 µL essential oil sample was injected and diluted inside the equipment (split, 50). The temperature of the injector was 200 °C. The initial oven temperature was 50 °C, maintained for 2 min, followed by an initial ramp-up of 10 °C/min up to 130 °C, the second ramp-up of 5 °C/min up to 150 °C, and the third ramp-up of 30 °C/min up to 190 °C, and held at the final temperature for 3 min. The main components, thymol and carvacrol, were identified by their retention time and the mass profile of the compounds available from the US National Institute of Standard Technology (NIST) library.

The pure chemicals thymol, and carvacrol were acquired from Sigma-Aldrich (St. Louis, MI, USA). The reference drug Amphotericin B (Amp B; in Vitro S.A., Mexico City, Mexico) was used as control of leishmanicidal activity.

The EOs, thymol, and carvacrol were dissolved in dimethylsulfoxide (DMSO; Sigma-Aldrich, St. Louis, MO, USA) to prepare a stock solution at 50 mg/mL concentration; the different dilutions for biological evaluation were made in the same cell culture medium at the time of use. DMSO did not exceed the final concentration of 1%, which is considered acceptable to avoid toxicity in the vertebrate host [49].

### 4.2. Parasites Culture

Parasite strain *L. mexicana* MNYC-BZ/62/M379 ATCC was used; the promastigotes were cultured at 27 °C in darkness in RPMI 1640 medium (Gibco, Carlsbad, CA, USA) supplemented with 10% heat-inactivated fetal bovine serum (FBS; Gibco, Grand Island, NY, USA) and 100 U/mL penicillin/streptomycin (In vitro S.A, Mexico City, Mexico) [39].

### 4.3. Cell Culture

Murine macrophage cell line J774A.1 (TIB-61 ATCC) was cultured in RPMI 1640 medium (Gibco, Carlsbad, CA, USA) supplemented with 10% heat-inactivated fetal bovine serum (FBS; Gibco, Grand Island, NY, USA), 1% MEM-NEAA medium (Gibco Invitrogen, Carlsbad, CA, USA) and 100 U/mL penicillin/streptomycin (In vitro S.A, Mexico City, Mexico) at 37 °C, humidity, and 5% CO_2_ [39].

### 4.4. In vitro Evaluation of Leishmanicidal Activity 

The metabolic inhibition in *L. mexicana* promastigotes was evaluated by the Alamar Blue (Invitrogen, Carlsbad, CA, USA) fluorometric method. Metacyclic parasites (7-day culture) were seeded (5×10^5^ parasites) in a 96-well plate in 100 µL of medium and treated with five different concentrations obtained by serial two-fold dilutions of the pure compounds and EOs (200–12.5 µg/mL). Amphotericin B (Amph B) was the reference drug and parasites in the presence of 0.5% DMSO (vehicle control) were the positive viability control. Each experiment was performed in triplicate.

After 24 h incubation, 10 µL of Alamar Blue (10% *v*/*v*) were added to each well and incubated for a further 24 h. The microplates were analyzed on a spectrophotometer (Spectramax Plus 384; Molecular Devices Corp, Sunnyvale, CA, USA) at 544 nm excitation and 590 nm emission. The absorbance of each treated well was compared with the vehicle control and was expressed as a percentage of metabolic inhibition; the 50% inhibitory concentration (IC_50_) was determined with probit statistical tool [17,39].

### 4.5. In vitro Evaluation of Cytotoxic Activity and Selectivity Index (SI)

Murine macrophages were seeded (5 × 10^4^ cells) in a 96-well plate and incubated for 24 h for monolayer formation. Subsequently, the concentration–response evaluation was carried out for the EOs, and the pure major components thymol and carvacrol were evaluated at different concentrations obtained by serial two-fold dilutions (200–12.5 µg/mL). As a negative cytotoxicity control, cells were incubated only in the presence of 0.5% DMSO. Each concentration was evaluated in triplicate. The microplate was incubated for 20 h, then 10 µL of Alamar Blue were added, and the microplate was left for an additional 4 h incubation. The metabolic inhibition analysis was performed as described previously in the leishmanicidal evaluation; finally, the cytotoxic concentration of 50% of the population (CC_50_) was obtained, and the selectivity index (SI) was calculated (CC_50_/IC_50_) [39,50].

### 4.6. Determination of Pharmacodynamic Interactions by Thymol and Carvacrol in L. mexicana

In a microplate, 5 × 10^5^ *L. mexicana* promastigotes were deposited, and combined microdilutions of different concentrations of thymol and carvacrol were added following the method defined as “checkerboard”. Combinations of different concentrations of each monoterpene starting from 200 µg/mL to 6.25 µg/mL (two-fold dilution series) were assayed. Alamar Blue was used to determine the viability or inhibition of each combination and the fractional inhibitory concentration (FIC) index was determined using the formula: FIC index = [A]/IC_50_A+ [B]/IC_50_B, where [A] and [B] are the IC_50_ values on promastigotes of monoterpenes in combination, and IC_50_A and IC_50_B are the IC_50_ values on the promastigotes of each monoterpene tested alone. CFI ≤ 0.5 is a synergistic effect, CFI > 0.5 and ≤1 is an additive effect, CFI > 1 and ≤2 is an indifferent effect, and CFI > 2.0 is an antagonistic effect [23].

### 4.7. Evaluation of Cell Death Markers on Promastigotes of L. mexicana

Promastigotes were treated with EOs, thymol, and carvacrol as described previously; after incubation, they were harvested, washed, and stained to carry out the corresponding analysis of cell death markers. Forward scatter (FSC) and side scatter (SSC) modifications, membrane permeability, annexin V binding, mitochondrial membrane potential, and DNA fragmentation were evaluated in a FACSCalibur flow cytometer (Becton Dickinson, San Jose, CA, USA) recording 10,000 events for each determination, and data were analyzed using the CellQuest Pro software version 6.1 (Becton Dickinson, San Jose, CA, USA).

#### 4.7.1. Forward Scatter (FSC) and Side Scatter (SSC) Modifications

Promastigotes treated and washed were suspended in PBS and acquired by flow cytometry. Changes in the size and complexity were analyzed by light dispersion shown in biparametric dot plots (FSC vs. SSC) [39].

#### 4.7.2. Membrane Permeability

The pellets of parasites were suspended in 300 μL of 0.4 μg/mL propidium iodide (PI; Sigma- Aldrich, St. Louis, MO, USA) in PBS and incubated 10 min at room temperature in darkness, and were subsequently acquired and analyzed by flow cytometry [39].

#### 4.7.3. Annexin V Binding 

Treated and washed promastigotes were stained using 3 µL Annexin V-FITC (BioLegend, San Diego, CA, USA) and incubated for 20 min in the dark at room temperature; the cells were suspended in 300 μL of annexin buffer. Data were obtained and analyzed by flow cytometry [39].

#### 4.7.4. Mitochondrial Membrane Potential (ψm)

After treatment whit the compounds, promastigotes were incubated for 30 min at room temperature in darkness with 20 μL of 10 μM Rhodamine 123 (Rho 123, Sigma, St. Louis, MO, USA), washed twice, and suspended in 300 μL of PBS. The parasites were acquired by flow cytometry [39].

#### 4.7.5. DNA Fragmentation

The parasite pellets harvested were treated with 500 μL of cold 70% ethanol and stored overnight at −20 °C to fix and permeabilize. Fixed promastigotes were washed twice with PBS and suspended in 300 μL of 50 μg/mL PI and 5 K units/mL RNAse (staining solution). Samples were incubated for an additional 30 min at room temperature in the dark. Parasites were acquired and analyzed by flow cytometry [39].

### 4.8. Morphology Analysis

Parasites treated were washed twice and resuspended in PBS, and then promastigotes were deposited onto slides using cytospin (LaboFuge 400, Thermo Scientific, USA). They were fixed with methanol and stained with Giemsa 1:10 diluted (Merck, Darmstadt, Germany) for 30 min, and the samples were examined on a light microscope at 40 and 100× (Primo Star, Zeiss). Images were captured with WiFi Digital Camera model MC4KW-G1 and analyzed with Micro Capture software version 7.9.

### 4.9. Statistical Analysis 

GraphPad Prism version 6.01 (GraphPad Software, San Diego, CA, USA) was utilized for statistical analysis. Two-way analysis of variance (ANOVA) was followed by Sidak’s or Tukey’s multiple comparisons test, as appropriate. Data are expressed as the mean ± standard error of the mean.

## 5. Conclusions 

The Mexican oregano EOs presented leishmanicidal activity, and their effect was attributed to the presence of thymol and carvacrol; however, their biological activity varies according to their composition. *Lippia berlandieri* EO had a better leishmanicidal effect than *Poliomintha longiflora* EO, and thymol was the pure compound with the best selectivity index. The mechanism of parasite death induced by thymol and carvacrol was apoptosis, with the same mechanism for both EOs. Mexican oregano EOs are potential sources of bioactive natural molecules for the research and development of alternative medicines for treating neglected diseases such as cutaneous leishmaniasis.

## Figures and Tables

**Table 1:** Leishmanicidal activity of Mexican oregano essential oils and their pure major compounds.

	*Leishmania mexicana* IC_50_ (µg/mL)	Macrophages J774 A.1 CC_50_ (µg/mL)	SI (CC_50_/CI_50_)
*Lippia berlandieri* EO	41.78 (36.48–47.09)	*150*.41 (144.03–156.77)	3.59
*Poliomintha longiflora* EO	79.70 (74.6–84.80)	166.66 (159.74–173.55)	2.01
Thymol	22.39 (16.77–20.01)	114.13 (108.45–119.80)	5.10
Carvacrol	61.52 (56.83–66.22)	147.37 (142.71–152.02)	2.39
Amphotericin B	0.41 (0.37–0.43)	112.45 (107.01–117.89)	276

The values in parentheses represent the confidence interval determined by the probit method with 95% confidence.

## Data Availability

Not applicable.

## Supplementary Materials

The following supporting information can be downloaded at: https://www.mdpi.com/article/10.3390/molecules27165183/s1, Table S1. Chemical compounds identified in the essential oils of *Lippia berlandieri* and *Poliomontha longiflora*; Figure S1: Characterization of the leishmanicidal activity of *Lippia berlandieri* EO by flow cytometry; Figure S2: Characterization of the leishmanicidal activity of *Poliomintha longiflora* EO by flow cytometry; Figure S3: Characterization of the leishmanicidal activity of thymol by flow cytometry; Figure S4: Characterization of the leishmanicidal activity of carvacrol by flow cytometry.

## References

[1] WHO Leishmaniasis. https://www.who.int/es/news-room/fact-sheets/detail/leishmaniasis.

[2] WHO (World Health Organization) WHO EMRO|Neglected Tropical Diseases|Health Topics. http://www.emro.who.int/health-topics/tropical-diseases/.

[3] Hotez P.J., Aksoy S., Brindley P.J., Kamhawi S. (2020). World neglected tropical diseases day. PLoS Negl. Trop. Dis..

[4] PAHO Leishmaniasis—OPS/OMS|Organización Panamericana De La Salud. https://www.paho.org/es/temas/leishmaniasis.

[5] Saito K., Murray H.W. (2020). Chiclero’s ulcer. Int. J. Infect. Dis..

[6] Monroy-Ostria A., Sanchez-Tejeda G., Clarborn D. (2016). Survey of Cutaneous Leishmaniasis in Mexico: Leishmania Species, Clinical Expressions and Risk Factors. The Epidemiology and Ecology of Leishmaniasis.

[7] Sundar S., Chakravarty J., Meena L.P. (2019). Leishmaniasis: Treatment, drug resistance and emerging therapies. Expert Opin. Orphan Drugs.

[8] Copeland N.K., Aronson N.E. (2015). Leishmaniasis: Treatment updates and clinical practice guidelines review. Curr. Opin. Infect. Dis..

[9] Juárez-Rosete C.R., Aguilar-Castillo J.A., Juárez-Rosete M.E., Bugarín-Montoya R., Juárez-López P., Cruz E. (2013). Hierbas aromáticas y medicinales en México: Tradición e innovación. Bio. Cienc..

[10] García-Pérez E., Castro-Álvarez F.F., Gutiérrez-Uribe J.A., García-Lara S. (2018). Revisión de la producción, composición fitoquímica y propiedades nutracéuticas del orégano mexicano. Rev. Mex. Cienc. Agrícolas.

[11] Cid-Pérez T.S., Ávila-Sosa R., Ochoa-Velasco C.E., Rivera-Chavira B.E., Nevárez-Moorillón G.V. (2019). Antioxidant and antimicrobial activity of mexican oregano (*Poliomintha longiflora*) essential oil, hydrosol and extracts fromwaste solid residues. Plants.

[12] Levario-Gómez A., Ávila-Sosa R., Gutiérrez-Méndez N., López-Malo A., Nevárez-Moorillón G.V. (2020). Modeling the Combined Effect of pH, Protein Content, and Mexican Oregano Essential Oil Against Food Spoilage Molds. Front. Sustain. Food Syst..

[13] Avila-Sosa R., Gastélum-Franco M.G., Camacho-Dávila A., Torres-Muñoz J.V., Nevárez-Moorillón G.V. (2010). Extracts of Mexican oregano (*Lippia berlandieri* Schauer) with antioxidant and antimicrobial activity. Food Bioprocess. Technol..

[14] Andrade-Ochoa S., Sánchez-Aldana D., Chacón-Vargas K.F., Rivera-Chavira B.E., Sánchez-Torres L.E., Camacho A.D., Nogueda-Torres B., Nevárez-Moorillón G.V. (2018). Oviposition deterrent and larvicidal and pupaecidal activity of seven essential oils and their major components against Culex quinquefasciatus say (Diptera: Culicidae): Synergism–antagonism effects. Insects.

[15] Portillo-Ruiz M.C., Viramontes-Ramos S., Muñoz-Castellanos L.N., Gastélum-Franco M.G., Virgnia N.-M.G. (2005). Antifungal Activity of Mexican Oregano (*Lippia berlandieri* Shauer). J. Food Prot..

[16] Zapién-Chavarría K.A., Plascencia-Terrazas A., Venegas-Ortega M.G., Varillas-Torres M., Rivera-Chavira B.E., Adame-Gallegos J.R., González-Rangel M.O., Nevárez-Moorillón G.V. (2019). Susceptibility of multidrug-resistant and biofilm-forming uropathogens to Mexican oregano essential oil. Antibiotics.

[17] Andrade-Ochoa S., Chacón-Vargas K.F., Sánchez-Torres L.E., Rivera-Chavira B.E., Nogueda-Torres B., Nevárez-Moorillón G.V. (2021). Differential antimicrobial effect of essential oils and their main components: Insights based on the cell membrane and external structure. Membranes.

[18] Adame-Gallegos J.R., Andrade-Ochoa S., Nevarez-Moorillon G.V. (2016). Potential Use of Mexican Oregano Essential Oil against Parasite, Fungal and Bacterial Pathogens. J. Essent. Oil-Bearing Plants.

[19] Oliveira V.C.S., Moura D.M.S., Lopes J.A.D., De Andrade P.P., Da Silva N.H., Figueiredo R.C.B.Q. (2009). Effects of essential oils from *Cymbopogon citratus* (DC) Stapf., *Lippia sidoides* Cham., and *Ocimum gratissimum* L. on growth and ultrastructure of Leishmania chagasi promastigotes. Parasitol. Res..

[20] Funari C.S., de Almeida L., Passalacqua T.G., Martínez I., Ambrosio D.L., Cicarelli R.M.B., Silva D.H.S., Graminha M.A.S. (2016). Oleanonic acid from *Lippia lupulina* (Verbenaceae) shows strong in vitro antileishmanial and antitrypanosomal activity. Acta Amaz..

[21] Escobar P., Leal S.M., Herrera L.V., Martinez J.R., Stashenko E. (2010). Chemical composition and antiprotozoal activities of Colombian Lippia spp essential oils and their major components. Mem. Inst. Oswaldo. Cruz..

[22] Weniger B., Robledo S., Arango G., Deharo E., Aragón R., Muñoz V., Callapa J., Lobstein A., Anton R. (2001). Antiprotozoal activities of Colombial plants. J. Ethnopharmacol..

[23] Pérez-Palma L.D., Andrade-Ochoa S., Levario-Gómez A., Cantú-Solis E., Sánchez-Aldana D., Nevárez-Moorillón G.V., Loya-Loya M.E. (2019). Evaluación de la propiedad antimicrobiana y antiadherente del sulfato de cobre (Cu2SO4) y monoterpenos sobre Lactobacillus acidophilus y Streptococcus mutans. Acta Univ..

[24] Kar A., Jayaraman A., Charan Raja M.R., Srinivasan S., Debnath J., Mahapatra S.K. (2021). Synergic effect of eugenol oleate with amphotericin B augments anti-leishmanial immune response in experimental visceral leishmaniasis in vitro and in vivo. Int. Immunopharmacol..

[25] de Carvalho R.d.C.V., de Sousa V.C., Santos L.P., dos Santos I.L., Diniz R.C., Rodrigues R.R.L., de Medeiros M.d.G.F., da F. Rodrigues K.A., de M. Alves M.M., Arcanjo D.D.R. (2021). Limonene-carvacrol: A combination of monoterpenes with enhanced antileishmanial activity. Toxicol. Vitr..

[26] Crowley L.C., Scott A.P., Marfell B.J., Boughaba J.A., Chojnowski G., Waterhouse N.J. (2016). Measuring cell death by propidium iodide uptake and flow cytometry. Cold Spring Harb. Protoc..

[27] Weingärtner A., Kemmer G., Müller F.D., Zampieri R.A., Gonzaga dos Santos M., Schiller J., Pomorski T.G. (2012). Leishmania promastigotes lack phosphatidylserine but bind annexin V upon permeabilization or miltefosine treatment. PLoS ONE.

[28] Ramakrishnan S., Serricchio M., Striepen B., Bütikofer P. (2013). Lipid synthesis in protozoan parasites: A comparison between kinetoplastids and apicomplexans. Prog. Lipid Res..

[29] Cid-Pérez T.S., Nevárez-Moorillón G.V., Torres-Muñoz J.V., Palou E., López-Malo A. (2016). Mexican Oregano (*Lippia Berlandieri* and *Poliomintha Longiflora*) Oils.

[30] de Melo J.O., Bitencourt T.A., Fachin A.L., Cruz E.M.O., de Jesus H.C.R., Alves P.B., de Fátima Arrigoni-Blank M., de Castro Franca S., Beleboni R.O., Fernandes R.P.M. (2013). Antidermatophytic and antileishmanial activities of essential oils from *Lippia gracilis* Schauer genotypes. Acta Trop..

[31] Farias-Junior A.P., Rios M.C., Moura T.A., Almeida R.P., Alves P.B., Blank A.F., Fernandes R.P.M., Scher R. (2012). Leishmanicidal activity of carvacrol-rich essential oil from *Lippia sidoides* cham. Biol. Res..

[32] de Medeiros M.d.G.F., da Silva A.C., das Graças Lopes Citó G.L., Borges A.R., de Lima S.G., Lopes J.A.D., Figueiredo R.C.B.Q. (2011). In vitro antileishmanial activity and cytotoxicity of essential oil from *Lippia sidoides* Cham. Parasitol. Int..

[33] Reza Youssefi M., Moghaddas E., Tabari A.M., Moghadamnia A.A., Hosseini S.M., Razieh B., Farash H., Ebrahimi M.A., Mousavi N.N., Fata A. (2019). In Vitro and In Vivo Effectiveness of Carvacrol, Thymol and Linalool against Leishmania infantum Mohammad. Molecules.

[34] Torres-Guerrero E., Quintanilla-Cedillo M.R., Ruiz-Esmenjaud J., Arenas R. (2017). Leishmaniasis: A review. F1000Research.

[35] Kachur K., Suntres Z. (2020). The antibacterial properties of phenolic isomers, carvacrol and thymol. Crit. Rev. Food Sci. Nutr..

[36] Anthony J.P., Fyfe L., Smith H. (2005). Plant active components—A resource for antiparasitic agents?. Trends Parasitol..

[37] Bassolé I.H.N., Juliani H.R. (2012). Essential oils in combination and their antimicrobial properties. Molecules.

[38] Ilaghi M., Sharifi I., Sharififar F., Sharifi F., Oliaee R.T., Babaei Z., Meimamandi M.S., Keyhani A., Bamorovat M. (2021). The potential role and apoptotic profile of three medicinal plant extracts on Leishmania tropica by MTT assay, macrophage model and flow cytometry analysis. Parasite Epidemiol. Control.

[39] Chacón-Vargas K.F., Andrade-Ochoa S., Nogueda-Torres B., Juárez-Ramírez D.C., Lara-Ramírez E.E., Mondragón-Flores R., Monge A., Rivera G., Sánchez-Torres L.E. (2018). Isopropyl quinoxaline-7-carboxylate 1,4-di-N-oxide derivatives induce regulated necrosis-like cell death on Leishmania (Leishmania) mexicana. Parasitol. Res..

[40] Basmaciyan L., Casanova M. (2019). Cell death in Leishmania. Parasite.

[41] Wlodkowic D., Telford W., Skommer J., Darzynkiewicz Z. (2011). Apoptosis and Beyond: Cytometry in Studies of Programmed Cell Death. Methods Cell Biol..

[42] Andrade Ochoa S., Chacón Vargas K.F., Correa Basuto J., Rodríguez Valdéz L.M., Nevarez-Moorillon G.V., Sanchez-Torres L., Méndez Vilas A. (2015). Rational design of new leishmanicidal agents: In silico and In vitro evaluation. The Battle against Microbial Pathogens: Basic Science, Technological and Educational Program.

[43] Monzote L., García M., Pastor J., Gil L., Scull R., Maes L., Cos P., Gille L. (2014). Essential oil from Chenopodium ambrosioides and main components: Activity against Leishmania, their mitochondria and other microorganisms. Exp. Parasitol..

[44] Le T.B., Beaufay C., Bonneau N., Mingeot-Leclercq M.-P., Quetin-Leclercq J. (2018). Anti-protozoal activity of essential oils and their constituents against Leishmania, Plasmodium and Trypanosoma Activité anti-protozoaire des huiles essentielles et de leurs constituants contre Leishmania, Plasmodium et Trypanosoma. Phytochimie.

[45] Monzote L., Alarcón O., Setzer W.N. (2012). Antiprotozoal activity of essential oils. Agric. Conspec. Sci..

[46] Machado M., Dinis A.M., Santos-Rosa M., Alves V., Salgueiro L., Cavaleiro C., Sousa M.C. (2014). Activity of Thymus capitellatus volatile extract, 1,8-cineole and borneol against Leishmania species. Vet. Parasitol..

[47] Luiz R.C., Cecchini A.L. (2021). Mitochondria as a Target for Monoterpenes.

[48] Monzote L., Pastor J., Scull R., Gille L. (2014). Antileishmanial activity of essential oil from Chenopodium ambrosioides and its main components against experimental cutaneous leishmaniasis in BALB/c mice. Phytomedicine.

[49] Galvao J., Davis B., Tilley M., Normando E., Duchen M.R., Cordeiro M.F. (2014). Unexpected low-dose toxicity of the universal solvent DMSO. FASEB J..

[50] Mahmoudvand H., Tavakoli R., Sharififar F., Minaie K., Ezatpour B., Jahanbakhsh S., Sharifi I. (2015). Leishmanicidal and cytotoxic activities of Nigella sativa and its active principle, thymoquinone. Pharm. Biol..

