# Supplementary material for: Mexican Oregano (Lippia berlandieri Schauer and Poliomintha longiflora Gray) Essential Oils Induce Cell Death by Apoptosis in Leishmania (Leishmania) mexicana Promastigotes
Source: Molecules. 2022 Aug 15;27(16):5183. doi: 10.3390/molecules27165183 (PMC9416784; doi:10.3390/molecules27165183)
Supplement: Supplementary file 1 [file molecules-27-05183-s001.zip › molecules-1858837-supplementary.pdf]

Article

Mexican Oregano (*Lippia berlandieri* Schauer and *Poliomintha longiflora* Gray) Essential Oils Induce Cell Death by Apoptosis in *Leishmania (Leishmania) mexicana* Promastigotes

Karla Fabiola Chacón-Vargas [1], Luvia Enid Sánchez-Torres [2,*], Mónica L. Chávez-González [3], Jaime R. Adame-Gallegos [1] and Guadalupe Virginia Nevárez-Moorillón [1,*]

Supplementary files

Table S1. Chemical compounds identified in the essential oils of *Lippia berlandieri* and *Poliomontha longiflora*

Compunds	%
***Lippia berlandieri* EO**	
Alpha-tuhjone	0.049
β-cis-Ocimene	0.09
Anisole	0.259
2,5-diethyl-3,6-dimethylpyrazine	0.64
Thymol	7.863
Carvacrol	33.781
M-cymen-4-ol	0.108
Phenol-2-methoxy (Guaiacol)	0.305
Adamantane,2-methyl	0.273
Gitoxigenin	0.941
Alpha-eudesmol	0.123
***Poliomintha longiflora* EO**	
β-pinene	0.637
Durene	21.064
Thymol	23.46
Carvacrol	18.35

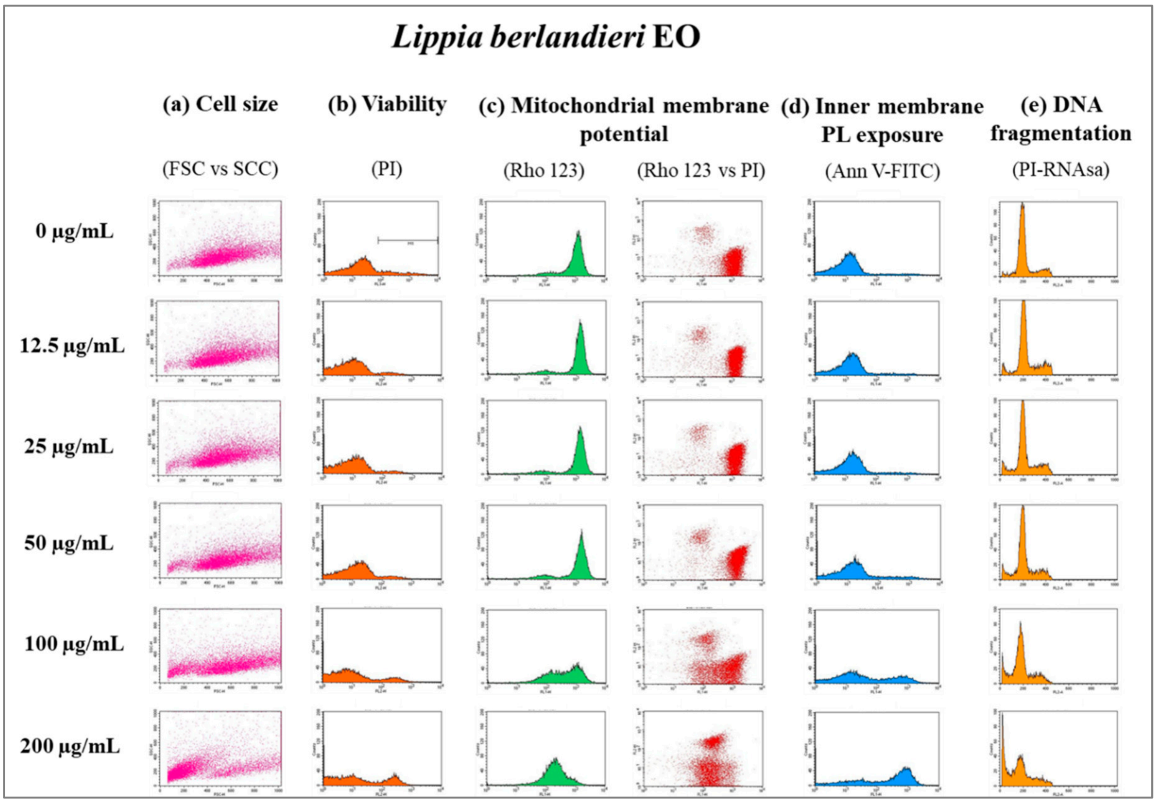

Figure S1. Characterization of the leishmanicidal activity of *Lippia berlandieri* EO by flow cytometry. *Leishmania mexicana* promastigotes were treated whit 0, 12.5, 25, 50, 100 and 200 µg/mL of *L. berlandieri* EO, incubated for 24 h and different markers of cell death were analyzed by flow cytometry. (**a**) Changes in size (FSC vs SSC); (**b**) Plasma membrane integrity; (**c**) Mitochondrial membrane potential (monoparametric and biparametric with PI analysis); (**d**) Inner membrane phospholipids (PL) exposure; (**e**) DNA fragmentation. The experiment was performed in triplicate, and representative images are shown.

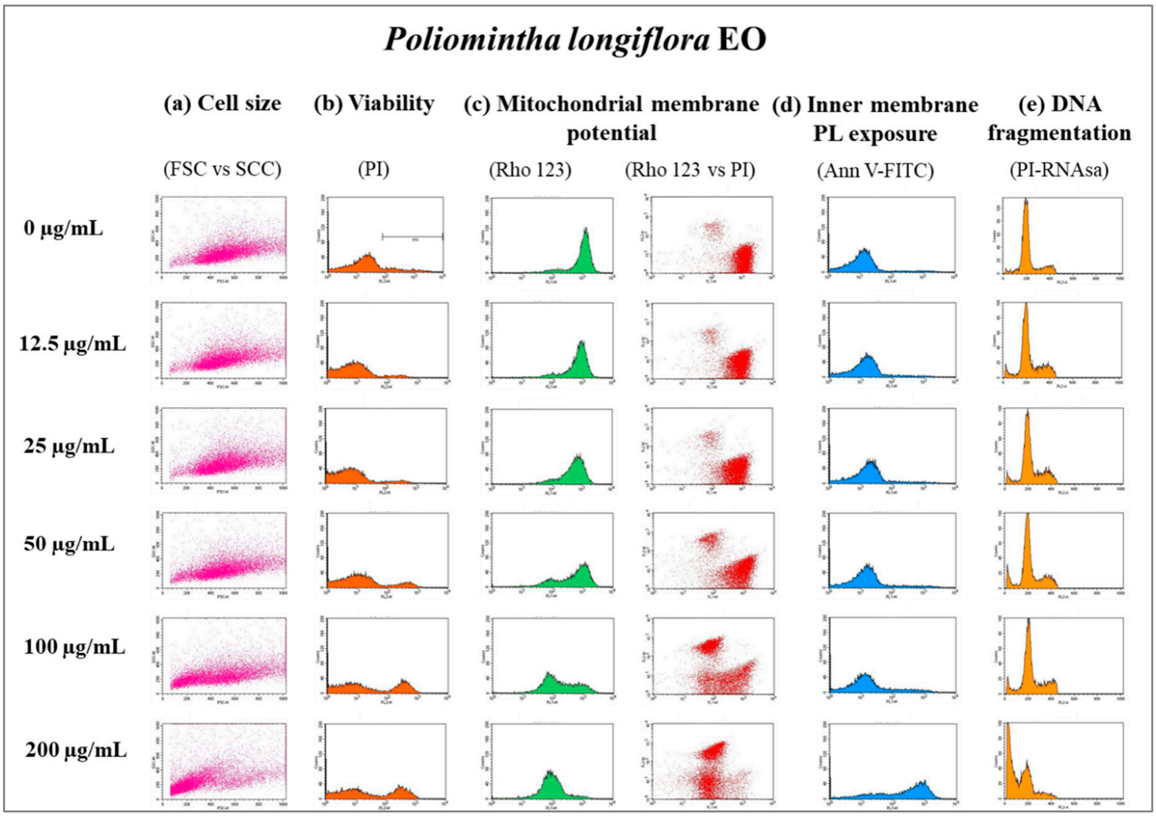

Figure S2. Characterization of the leishmanicidal activity of *Poliomintha longiflora* EO by flow cytometry. *Leishmania mexicana* promastigotes were treated whit 0, 12.5, 25, 50, 100 and 200 µg/mL of *P. longiflora* EO, incubated for 24 h and different markers of cell death were analyzed by flow cytometry. (**a**) Changes in size (FSC vs SSC); (**b**) Plasma membrane integrity; (**c**) Mitochondrial membrane potential (monoparametric and biparametric with PI analysis); (**d**) Inner membrane phospholipids (PL) exposure; (**e**) DNA fragmentation. The experiment was performed in triplicate, and representative images are shown.

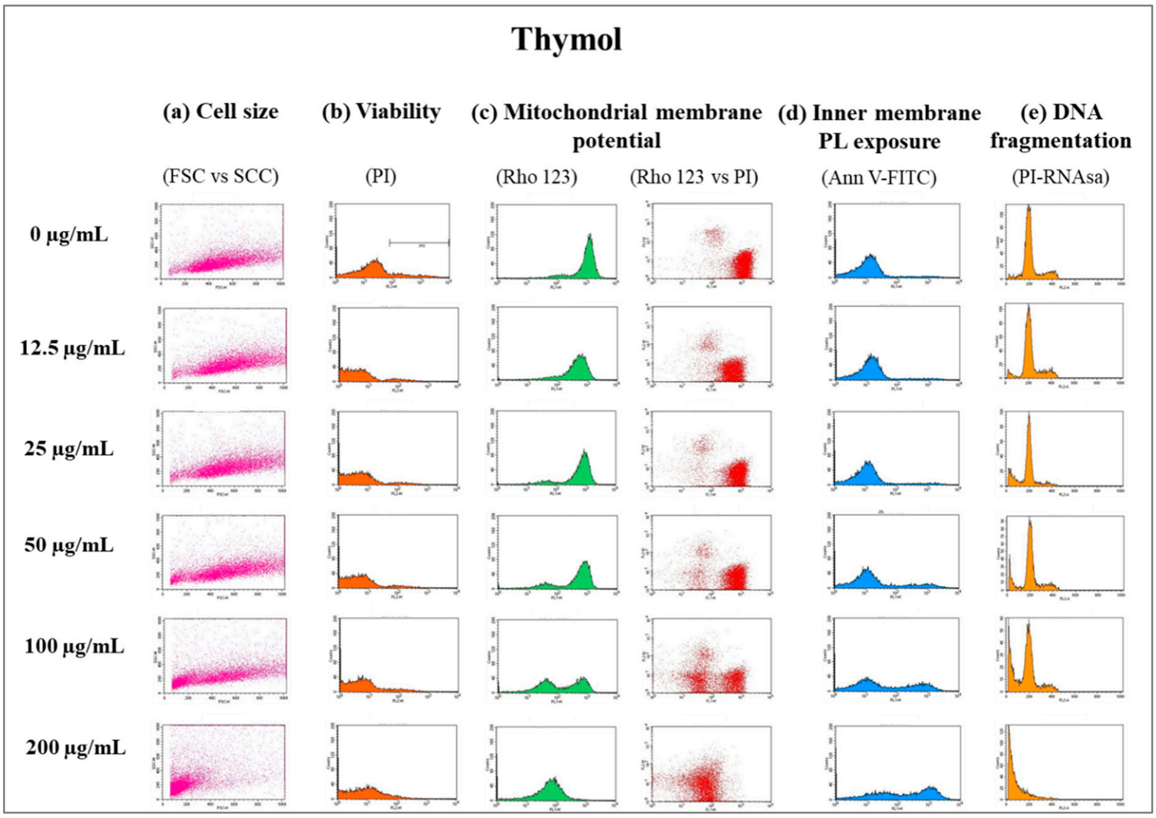

Figure S3. Characterization of the leishmanicidal activity of thymol by flow cytometry. *Leishmania mexicana* promastigotes were treated whit 0, 12.5, 25, 50, 100 and 200 µg/mL of thymol incubated for 24 h and different markers of cell death were analyzed by flow cytometry. a) Changes in size (FSC vs SSC); b) Plasma membrane integrity; d) Mitochondrial membrane potential (monoparametric and biparametric with PI analysis); c) Inner membrane phospholipids (PL) exposure; (e) DNA fragmentation. The experiment was performed in triplicate, and representative images are shown.

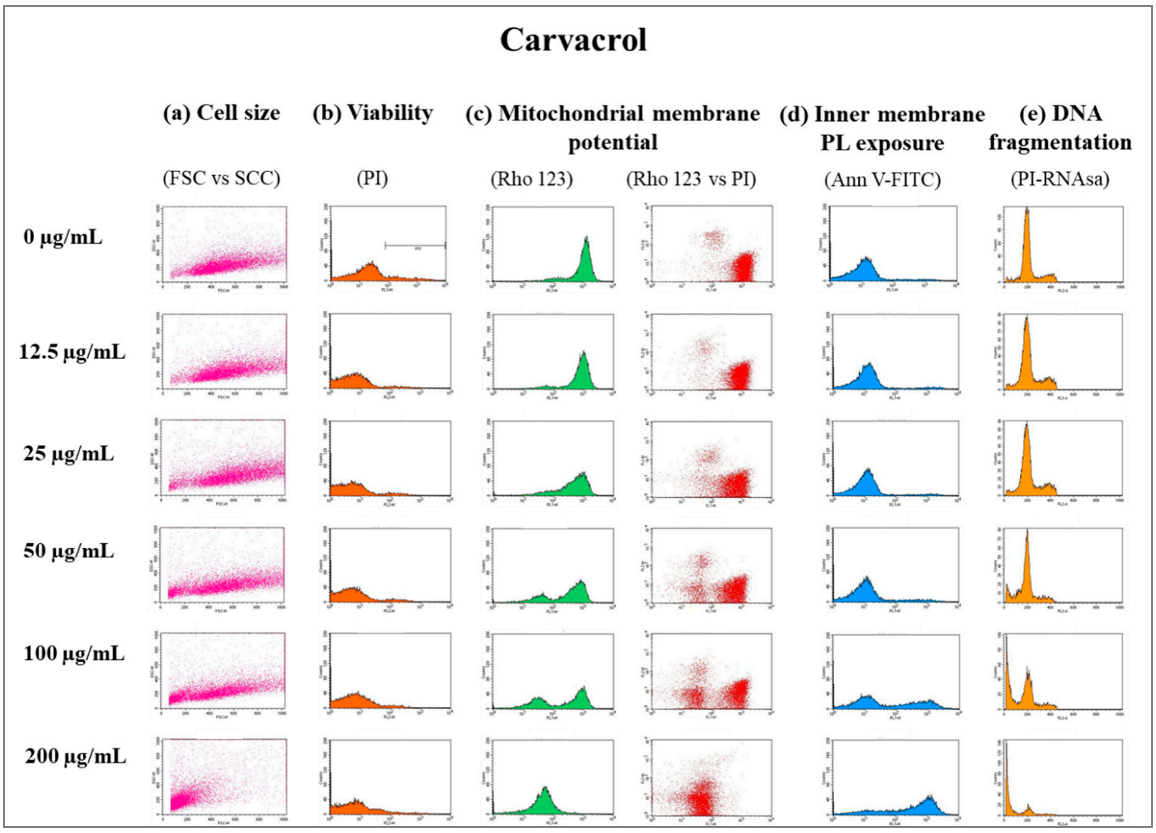

Figure S4. Characterization of the leishmanicidal activity of carvacrol by flow cytometry. *Leishmania mexicana* promastigotes were treated whit 0, 12.5, 25, 50, 100 and 200 µg/mL of carvacrol, incubated for 24 h and different markers of cell death were analyzed by flow cytometry. a) Changes in size (FSC vs SSC); b) Plasma membrane integrity; d) Mitochondrial membrane potential (monoparametric and biparametric with PI analysis); c) Inner membrane phospholipids (PL) exposure; (e) DNA fragmentation. The experiment was performed in triplicate, and representative images are shown.
